# Proinflammatory cytokine TNFα promotes HPV-associated oral carcinogenesis by increasing cancer stemness

**DOI:** 10.1038/s41368-019-0069-7

**Published:** 2020-01-07

**Authors:** Hannah S. Hong, Jonathan Akhavan, Sung Hee Lee, Reuben H. Kim, Mo K. Kang, No-Hee Park, Ki-Hyuk Shin

**Affiliations:** 1 The Shapiro Family Laboratory of Viral Oncology and Aging Research, UCLA School of Dentistry, Los Angeles, CA USA; 2 UCLA Jonsson Comprehensive Cancer Center, Los Angeles, CA USA; 3 Department of Medicine, David Geffen School of Medicine at UCLA, Los Angeles, CA USA

**Keywords:** Cancer stem cells, Oral cancer

## Abstract

High-risk human papillomaviruses (HPVs) are involved in the development of several human cancers, including oropharyngeal squamous cell carcinomas. However, many studies have demonstrated that HPV alone is not sufficient for the oncogenic transformation of normal human epithelial cells, indicating that additional cofactors are required for the oncogenic conversion of HPV-infected cells. Inasmuch as chronic inflammation is also closely associated with carcinogenesis, we investigated the effect of chronic exposure to tumor necrosis factor α (TNFα), the major proinflammatory cytokine, on oncogenesis in two immortalized oral keratinocyte cell lines, namely, HPV16-immortalized and human telomerase reverse transcriptase (hTERT)-immortalized cells. TNFα treatment led to the acquisition of malignant growth properties in HPV16-immortalized cells, such as (1) calcium resistance, (2) anchorage independence, and (3) increased cell proliferation in vivo. Moreover, TNFα increased the cancer stem cell-like population and stemness phenotype in HPV16-immortalized cells. However, such transforming effects were not observed in hTERT-immortalized cells, suggesting an HPV-specific role in TNFα-promoted oncogenesis. We also generated hTERT-immortalized cells that express HPV16 E6 and E7. Chronic TNFα exposure successfully induced the malignant growth and stemness phenotype in the E6-expressing cells but not in the control and E7-expressing cells. We further demonstrated that HPV16 E6 played a key role in TNFα-induced cancer stemness *via* suppression of the stemness-inhibiting microRNAs miR-203 and miR-200c. Overexpression of miR-203 and miR-200c suppressed cancer stemness in TNFα-treated HPV16-immortalized cells. Overall, our study suggests that chronic inflammation promotes cancer stemness in HPV-infected cells, thereby promoting HPV-associated oral carcinogenesis.

## Introduction

High-risk human papillomaviruses (HPVs) infection is closely associated with the development of female genital epithelial cancers. Over 90% of cervical cancer biopsies contain high-risk HPV DNA, such as HPV-16 and HPV-18 DNA.^1^ Many studies have also revealed that HPV is an additional independent risk factor for a subset of head and neck squamous cell carcinoma (HNSCC) patients.^2–5^ Similarly, HPV infection is also closely linked to malignant oral lesions.^6^ Transformation of normal foreskin and exocervical, cervical, and oral epithelial cells, which are the primary in vivo target cells of HPV infection, has been achieved by cloned high-risk HPV DNA.^7–9^ These transformed cells are, in general, immortal, anchorage-dependent in vitro, and nontumourigenic in nude mice.^7–9^ Moreover, most human cervical squamous epithelium containing high-risk HPVs does not progress to in situ or invasive carcinomas.^10,11^ This may implicate environmental and genetic cofactors as necessary for the malignant conversion of HPV-infected cells. The ubiquity of HPV infections, the regression of most HPV-induced dysplasias, and the long incubation period between initial infection and development of cancer indicate that HPV infection by itself may not be sufficient for neoplastic conversion of normal epithelial cells.^12^

There is increasing evidence of chronic inflammation-associated tumourigenesis.^13^ Although the molecular and cellular mechanisms linking chronic inflammation to tumourigenesis have not been fully understood, tumor necrosis factor α, TNFα, a major mediator of inflammation, plays a crucial role in inflammation-associated cancer development. Disruption of the TNFα signaling pathway could significantly inhibit chemical-induced carcinogenesis in the skin.^14,15^ Many studies have suggested that TNFα promotes inflammation-associated tumourigenesis by activating nuclear factor-κB signaling,^16,17^ which inhibits the death of precancerous or transformed cells during the development of inflammation-associated cancers.^18–20^ In addition, TNFα has been shown to be a potential mutagen that causes DNA damage through the induction of reactive oxygen species.^21^

Chronic inflammation has been identified as a cofactor for HPV-associated cervical carcinogenesis.^22,23^ However, the precise mechanism by which chronic inflammation might contribute to HPV-associated tumourigenesis is unclear. TNFα has been shown to promote HPV gene expression in HPV-immortalized keratinocytes. This suggests that TNFα contributes to HPV persistence and subsequent neoplastic progression by increasing viral gene expression.^24,25^ TNFα increases the proliferation of HPV-immortalized and HPV-infected cervical cancer cell lines by upregulating amphiregulin, an epidermal growth factor receptor ligand.^26^ These data suggest that TNFα and HPV may act in concert to induce neoplastic cell growth within the squamous epithelium infected with HPV, leading to the development of secondary molecular and genetic events, which eventually result in malignant conversion.^21^ However, most of the molecular events underlying the co-carcinogenic role of TNFα in HPV-associated carcinogenesis remain elusive and warrant further study.^27,28^

Recent studies have uncovered and validated the pathophysiologic role of self-renewing cells, namely, cancer stem cells (CSCs; also known as tumor-initiating cells), in long-term maintenance of cancers.^29^ CSCs share many molecular similarities to embryonic and normal adult stem cells. Many molecular determinants of normal stem cells, such as self-renewal ability and multi-lineage differentiation capacity, are retained in CSCs.^29^ CSCs have been isolated from various primary tumors and established cancer cell lines via cell surface markers, and they typically have the following properties: high tumourigenicity upon injection in immunodeficient mice, the ability to grow as tumor spheres in undifferentiating medium and resistance to cancer therapeutic agents.^29^ Therefore, CSCs drive the perpetuity of the disease while producing cellular heterogeneity of cancer tissues and are becoming new targets of anticancer therapies.^29^ The phenotypes of CSCs have been reported to be maintained by several endogenous signaling pathways, including Notch, Hedgehog, and Wnt.^30^ In addition to the endogenous pathways, CSCs could be enriched by exogenous carcinogenic factors. For instance, nicotine has been shown to increase the aldehyde dehydrogenase (ALDH)-positive CSC population in human breast cancer *via* a Notch-dependent pathway.^31^ Furthermore, recent studies have demonstrated that the proinflammatory cytokines TGFβ and TNFα generate CSCs in human cancer.^32–34^ In the present study, we investigated the effect of chronic inflammation on HPV-associated oral carcinogenesis by treating HPV-immortalized and non-tumourigenic human oral keratinocytes with TNFα for extended periods and studied the phenotypic and molecular biological changes.

## Results

Chronic TNFα exposure induces calcium resistance in HPV-immortalized cells but not in non-HPV-immortalized cells.

**Fig. 1:**
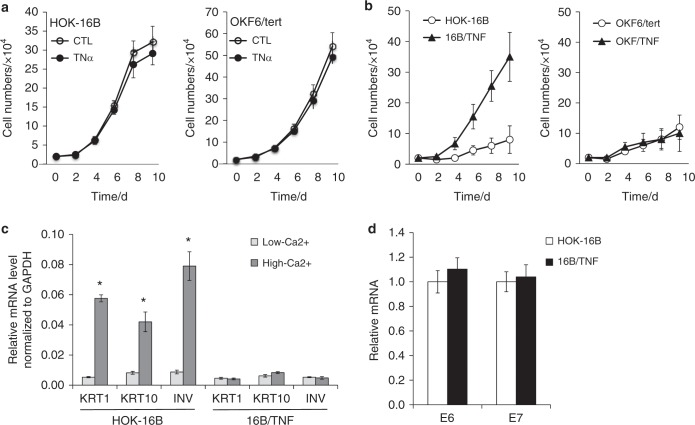
Chronic TNFα exposure induces calcium resistance in HPV-immortalized oral keratinocytes. **a** HPV16-immortalized HOK-16B and hTERT-immortalized OKF6/tert cells were exposed to TNFα (5 ng·mL^−1^) in low-Ca^2+^ (0.15 mmol·L^−1^) keratinocyte growth medium (KGM) for the indicated days, and the cell numbers were counted. **b** HOK-16B and OKF6/tert cells were exposed to TNFα (5 ng·mL^−1^) for 4 months in low-Ca^2+^ medium to generate 16B/TNF and OKF/TNF cells, respectively. Then, the cell proliferation capacity in high-Ca^2+^ (1.5 mmol·L^−1^) DMEM containing 10% serum was determined by cell counting. Cells were seeded at a density of 2 × 10^4^ cells and counted after the indicated incubation period. Passage-matched controls, HOK-16B and OKF6/tert cells, were used for comparison with 16B/TNF and OKF/TNF cells, respectively. **c** The effect of high Ca^2+^ on the expression of differentiation markers was determined by qPCR using HOK-16B and 16B/TNF cells. The cells were cultured in low- or high-Ca^2+^ medium for 2 days and harvested for the assay. *P *<* 0.01 compared to the low-Ca^2^ group by two-tailed Student’s *t* test. **d** Effect of chronic TNFα exposure on the expression of HPV16 E6 and E7 was determined by qPCR using HOK-16B and 16B/TNF cells.

Two immortalized oral keratinocyte cell lines (HPV16-immortalized HOK-16B and hTERT-immortalized OKF6/tert) were used in this study. Keratinocytes normally proliferate in low-Ca^2+^ (0.15 mmol·L^−1^) keratinocyte growth medium (KGM) but not in high-Ca^2+^ (1.5 mmol·L^−1^) DMEM containing 10% serum. Proliferation capacity at the physiological calcium level (1.5 mmol·L^−1^), also known as calcium resistance, is a transformed phenotype of keratinocytes.^35^ To investigate the effect of inflammation on HPV-associated carcinogenesis, we first examined the effect of short-term proinflammatory cytokine TNFα exposure (2–10 days) on the proliferation of HPV-positive HOK-16B and HPV-negative OKF6/tert cells in low-Ca^2+^ medium (Fig. 1a). The short-term TNFα exposure had no significant effect on cell growth. Interestingly, after 4 months of exposure to TNFα, HOK-16B cells showed enhanced proliferation capacity in the high-Ca^2+^ medium and no signs of keratinocyte differentiation and cell death; they were named 16B/TNF (Fig. 1b). However, after the same period of exposure, OKF6/tert cells failed to show enhanced proliferation capacity in the high-Ca^2+^ medium and were named OKF/TNF (Fig. 1b). Moreover, high Ca^2+^ markedly increased the expression of differentiation markers, i.e., keratin 1 (KRT1), KRT10, and involucrin (INV), in HOK-16B but not in 16B/TNF cells (Fig. 1c). Our data indicate that chronic TNFα treatment resulted in calcium resistance and a significant reduction in the differentiation potential of the HPV-positive HOK-16B cells. Since TNFα is known to affect HPV viral gene expression,^24^ we measured the expression levels of E6 and E7 in HOK-16B and 16B/TNF cells (Fig. 1d). E6 and E7 expression levels were not altered by TNFα in the HPV16-immortalized oral keratinocytes. Collectively, our findings suggest that the acquired calcium resistance of 16B/TNF cells is independent of the overexpression of E6/E7 by TNFα in HPV16-immortalized oral keratinocytes.

Chronic TNFα exposure induces malignant growth properties in HPV-immortalized cells but not in non-HPV-immortalized cells.

**Fig. 2:**
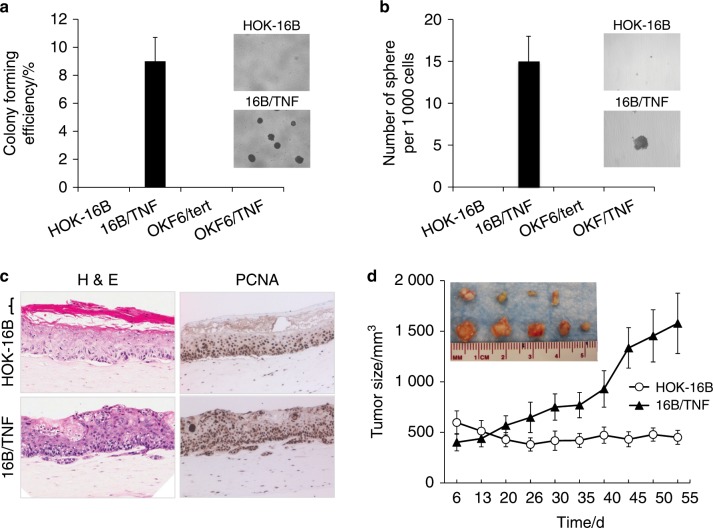
Chronic TNFα exposure induces malignant growth properties in HPV-immortalized oral keratinocytes. a Anchorage-independent growth ability was determined by soft agar assay. Five thousand cells were seeded on 0.4% soft agar and incubated for 3 weeks. Colonies were counted, and images were acquired at a magnification of 40×. The assay was performed in the absence of TNFα. **b** Self-renewal capacity was determined by tumor sphere formation assay. Single cells were plated in ultralow attachment plates at a density of 1 000 cells per mL in serum-free tumor sphere-forming medium. Tumor spheres were counted on day 17, and images were acquired at a magnification of ×40. The organotypic raft assay was performed in the absence of TNFα. **c** Organotypic raft cultures were established with HOK-16B and 16B/TNF cells. After 14 days of air lifting, the mucosal tissue constructs were harvested and processed for H&E staining and immunohistochemical staining against PCNA. Bracket indicates a terminally differentiated cornified layer, which is missing in the raft culture of 16B/TNF cells. Slides were scanned at ×40 magnification. The organotypic raft assay was performed in the absence of TNFα. **d** In vivo tumourigenicity was determined by xenograft tumor assay. HOK-16B and 16B/TNF cells were injected subcutaneously into five nude mice.

We further examined the effect of chronic TNFα exposure on malignant growth properties, such as anchorage independence and self-renewal. A soft agar assay revealed that only 16B/TNF cells acquired anchorage-independent growth ability (Fig. 2a). A tumor sphere formation assay showed that 16B/TNF cells drastically increased self-renewal capacity as evinced by robust tumor sphere formation, while HOK-16B cells failed to form spheres (Fig. 2b). However, such growth properties were not observed in OKF6/tert and OKF/TNF cells. We also evaluated the epithelial formation and differentiation potential of HOK-16B and 16B/TNF cells in organotypic raft culture where we reconstituted squamous epithelium.^34,36^ 16B/TNF cells formed an irregular epithelial sheath showing increased epithelial thickness and invasion into the subepithelial layer (Fig. 2c). Moreover, 16B/TNF cells showed an increase in the expression of proliferating cell nuclear antigen (PCNA), a marker of cell proliferation, compared to the control (Fig. 2c). 16B/TNF cells failed to form a terminally differentiated cornified layer, while HOK-16B cells were able to form a terminally differentiated layer in the organotypic culture (Fig. 2c). These data indicate that chronic TNFα treatment led to not only a reduction in differentiation potential but also an increase in aberrant proliferation. To examine the in vivo tumourigenic potential, we injected the cells into nude mice and observed tumor formation. All (5/5) mice injected with 16B/TNF cells developed palpable nodules, and 4 out of 5 mice injected with HOK-16B cells formed nodules (Fig. 2d). However, 16B/TNF cells developed nodules faster than HOK-16B, and the sizes of the nodules were much greater than those developed from HOK-16B cells (Fig. 2d). Histological examination revealed that cystic nodules were formed by HOK-16B cells (data not shown). Nevertheless, the nodules developed from 16B/TNF cells showed increased cell proliferation in vivo compared to those from HOK-16B and displayed keratin pearls (data not shown). Since the presence of keratin pearls is important in the histologic diagnosis of squamous cell carcinoma,^37^ our data indicate that chronic TNFα exposure increases the in vivo growth capacity of HOK-16B cells. Taken together, our data indicate that TNFα induces malignant growth properties in HPV-immortalized keratinocytes but not in non-HPV-immortalized keratinocytes. It should be noted that after a 4-month exposure, we withdrew TNFα from the culture medium and performed all experiments in the absence of TNFα. In doing so, we could exclude the immediate effect of TNFα on the biological behaviors of the tested cell lines.

### Chronic TNFα exposure increases the CSC phenotype in HPV-immortalized cells

**Fig. 3:**
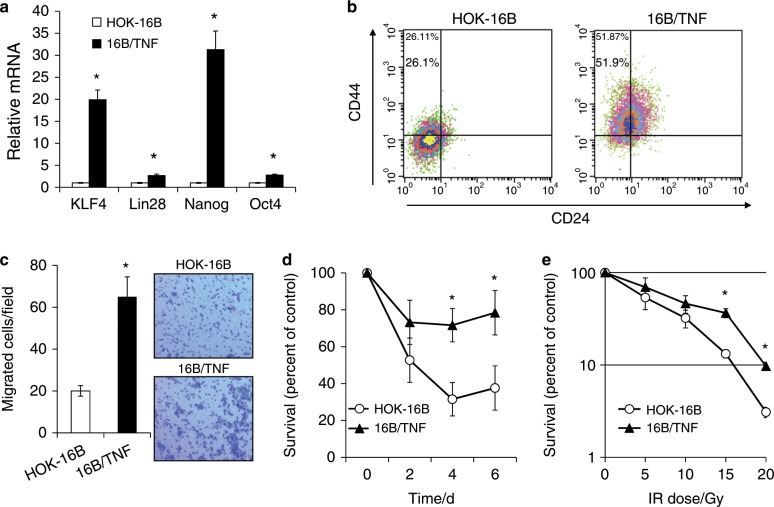
Chronic TNFα exposure increases the CSC phenotype in HPV-immortalized oral keratinocytes. **a** Levels of key stemness transcription factors, KLF4, Lin28, Nanog and Oct4, were measured by qPCR and normalized to the expression of GAPDH. *P *<* 0.01 compared to HOK-16B cells by two-tailed Student’s *t* test. **b** CD44 and CD24 stem cell surface markers were measured by flow cytometry analysis. Dots that fell in the upper left quadrant represent the CD44^high^/CD24^low^ CSC population. **c** Migration ability was determined by transwell migration assay. Representative images of the transwell migration assay are shown on the right. *P *<* 0.01. The assay was performed in the absence of TNFα. **d** Chemosensitivity assay. Five hundred cells were seeded in 96-well plates and treated with 40 µmol·L^−1^ cisplatin. At each incubation period, cell viability was measured using the MTT assay. *P *<* 0.05. **e** Radiosensitivity assay. Two hundred cells were seeded in 6-well plates and irradiated with different doses. After 10 days, surviving colonies were stained and counted. The assays were performed in the absence of TNFα.

Since chronic exposure to TNFα conferred self-renewal capacity, the key feature of CSCs,^29^ to HPV-immortalized oral keratinocytes, we investigated the effect of chronic TNFα exposure on the CSC phenotype in HOK-16B cells. Key stemness transcription factors (i.e., KLF4, Lin28, Nanog, and Oct4) were consistently increased in 16B/TNF compared to control HOK-16B cells (Fig. 3a). However, OKF/TNF failed to show such changes (data not shown). CSCs are also known to have the following properties: stem cell surface marker expression (e.g., CD44^high^/CD24^low^), high motility, and resistance to cancer therapeutic agents.^29^ There was a significant increase in the CD44^high^/CD24^low^ CSC population in 16B/TNF compared to HOK-16B cells (51.87% vs. 26.11%; Fig. 3b). As demonstrated by a transwell migration assay (Fig. 3c), 16B/TNF cells migrated 3 times faster than HOK-16B cells. Furthermore, 16B/TNF cells were more resistant to cisplatin (Fig. 3d) and ionizing radiation (Fig. 3e) than HOK-16B cells. Overall, these results indicate that chronic TNFα exposure resulted in an increase in the CSC population and properties in the HPV-immortalized keratinocytes, suggesting that chronic inflammation promotes HPV-associated oral carcinogenesis by increasing the stemness of HPV-infected cells.

### Chronic TNFα exposure induces malignant growth and CSC phenotype in non-HPV-immortalized cells expressing HPV16 E6

**Fig. 4:**
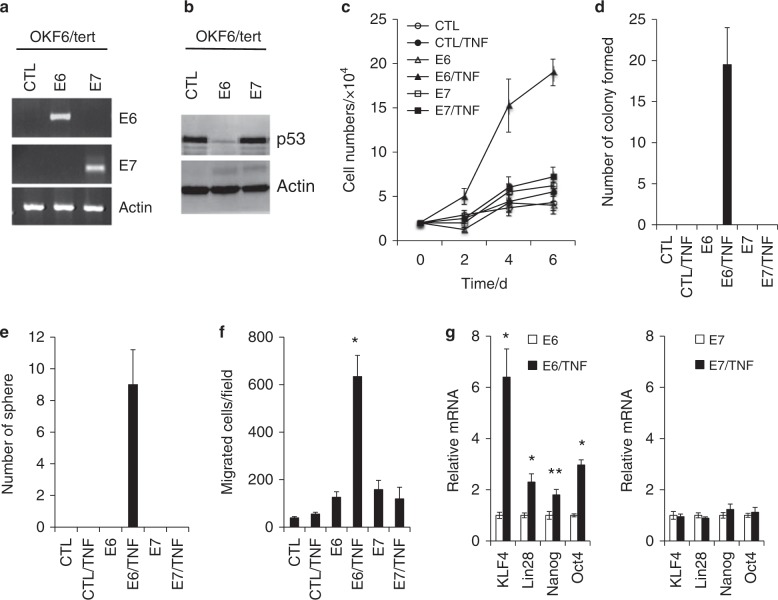
Chronic TNFα exposure induces malignant growth and CSC phenotype in OKF6/tert cells expressing HPV16 E6. HPV16 E6 and E7 expression was transduced into hTERT-immortalized OKF6/tert cells by infection with retroviral vectors or empty vector (CTL) as a control. CTL: OKF6/tert cells transfected with empty vector, E6: OKF6/tert cells expressing E6, E7: OKF6/tert cells expressing E6. **a** Ectopic expression of HPV16 E6 and E7 was confirmed by qPCR. **b** Expression of p53 in OKF6/tert cells expressing E6 and E7 was determined by Western blot analysis. Actin was used as a loading control. **c** CTL, E6 and E7 cells were exposed to TNFα (5 ng·mL^−1^) for 4 months to generate CTL/TNF, E6/TNF and E7/TNF cells, respectively. Proliferation capacity in medium with physiological levels of Ca^2+^ (1.5 mmol·L^−1^) was determined by cell counting. **d** Anchorage-independent growth ability was determined by soft agar assay. **e** Self-renewal capacity was determined by a tumor sph**e**re formation assay. **f** Migration ability was determined by transwell migration assay. *P *<* 0.01 compared to E6 by two-tailed Student’s *t* test. **g** Levels of key stemness transcription factors, KLF4, Lin28, Nano**g** and Oct4, were measured by qPCR and normalized to the expression of GAPDH. *P *<* 0.01 and **P *<* 0.05.

To further test the HPV-specific role in TNFα-promoted oral carcinogenesis, HPV16 E6 or E7 was transduced into the HPV-negative OKF6/tert cells through a retroviral vector, and empty vector was used as a control (Fig. 4a). The p53 protein level was reduced in the E6-expressing OKF6/tert cells, confirming the active expression of the E6 protein (Fig. 4b). Using these newly established isogenic OKF6/tert cells, we investigated the HPV-specific effects on TNFα-induced malignant growth and CSC phenotype. Chronic TNFα exposure induced calcium resistance (Fig. 4c) and anchorage-independent growth ability (Fig. 4d) in the E6-expressing cells but not in the control and E7-expressing cells. TNFα exposure also induced CSC properties, such as self-renewal capacity (Fig. 4e) and migration ability (Fig. 4f), in the E6-expressing cells. Moreover, the key stemness transcription factors (i.e., KLF4, Lin28, Nanog, and Oct4) were upregulated in E6/TNF cells compared to their corresponding control E6 cells (Fig. 4g). However, such alterations were not observed in the E7 and E7/TNF cells. These data indicate that HPV16 E6 plays a crucial role in TNFα-induced malignant growth and cancer stemness, confirming the HPV-specific role in inflammation-associated oral carcinogenesis.

### Chronic TNFα exposure suppresses stemness-inhibiting miRNAs in HPV-immortalized cells

**Fig. 5:**
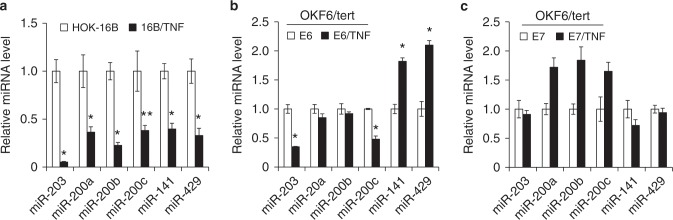
Chronic TNFα exposure decreases stemness-inhibiting miRNAs in HPV-immortalized cells and E6-expressing cells. **a** Differentially expressed stemness-inhibiting miRNAs (i.e., miR-203, miR-200a, miR-200b, miR-200c, miR-141, miR-429) in microarray data were validated by qPCR using HOK-16B and 16B/TNF cells. Levels of miRNAs were normalized to the expression of U6 snRNA. *P *<* 0.01 and **P *<* 0.05. **b** Levels of the stemness-inhibiting miRNAs were compared in the OKF6/tert cells expressing E6 and E6/TNF cells. **c** Levels of the stemness-inhibiting miRNAs were compared in the OKF6/tert cells expressing E7 and E7/TNF cells.

miRNAs are epigenetic regulators of gene expression, and their deregulation plays an important role in cancer stemness.^38^ To understand the role of miRNAs in the TNFα-induced cancer stemness of HPV-immortalized keratinocytes, we compared the global miRNA expression profiles of 16B/TNF with HOK-16B cells by using the miRCURY LNA^TM^ miRNA Array (Exiqon). The assay revealed that stemness-related miRNAs were frequently dysregulated in 16B/TNF cells (data not shown). As validated by qPCR (Fig. 5a), stemness-inhibiting miRNAs, e.g., miR-203 and the miR-200 family (miR-200a, 200b, 200c, 141, and 429)^39,40^ were markedly decreased in 16B/TNF compared to HOK-16B cells. Normal and cancer stem cells have reduced expression of miR-200 family members and miR-203, which results in increased expression of the stem cell factors.^41^ Moreover, miR-203 and miR-200c were found to be downregulated by chronic TNFα exposure in the OKF6/tert cells expressing E6 (Fig. 5b) but not in the cells expressing E7 (Fig. 5c), suggesting a role for the E6/miR-203 and/or miR-200c axis in the TNFα-induced CSC phenotype.

### Ectopic expression of miR-203 and miR-200c suppresses CSC properties in TNFα-treated HPV-immortalized oral keratinocytes

**Fig. 6:**
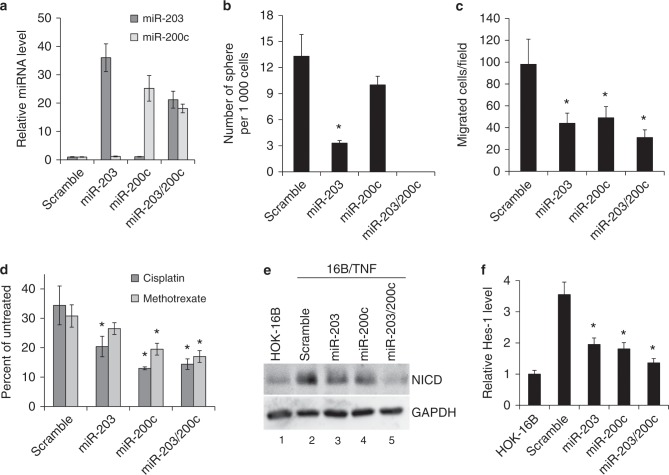
miR-203 and miR-200c suppress the CSC properties of TNFα-treated HPV-immortalized oral keratinocytes. **a** qPCR analysis of ectopic overexpression of miR-203 and miR-200c in 16B/TNF cells transfected with pre-miR-203, pre-miR-200c, or a combination of both miRNAs. Scramble oligonucleotides were transfected into 16B/TNF cells as a control. The relative amount of miRNAs in 16B/TNF cells transfected with pre-miRNAs was plotted as fold induction compared to that in 16B/TNF cells transfected with the scramble control 6 days post transfection. **b** The effect of miR-203 and miR-200c on the self-renewal of 16B/TNF cells was determined by a tumor sphere formation assay. *P *<* 0.01 compared to the scramble control by two-tailed Student’s *t* test. **c** Effect of miR-203 and miR-200c on migration of 16B/TNF cells was determined by transwell migration assay. *P *<* 0.05. **d** Effect of miR-203 and miR-200c on chemoresistance of 16B/TNF cells was determined by chemosensitivity assay. Five hundred cells were seeded in 96-well plates and treated with 40 µmol·L^−1^cisplatin (Cisp) or 25 µmol·L^−1^ methotrexate (Meth) for 4 days. *P *<* 0.05 compared to scramble. **e** The effect of miR-203 and miR-200c on the Notch pathway was determined by Western blotting using an antibody against the activated form of Notch1 protein (NICD). **f** Effect of miR-203 and miR-200c on the NICD downstream target Hes-1 was determined by qPCR. *P *<* 0.05 compared to the scramble control.

To understand the functional roles of miR-203 and miR-200c in TNFα-induced CSC properties, we ectopically expressed these miRNAs in 16B/TNF cells by transfecting them with pre-miR-200c, pre-miR-203 or a combination of both pre-miRs. Mature miR-200c and miR-203 were highly expressed in 16B/TNF cells transfected with the corresponding pre-miR, while the expression level of both miRNAs was not altered in 16B/TNF cells transfected with scramble control oligonucleotides (Fig. 6a). The expression of miR-203 and miR-200c decreased the self-renewal capacity of 16B/TNF cells (Fig. 6b). The combination of miR-203 and miR-200c had the strongest inhibitory effect on the tumor sphere-forming ability of 16B/TNF cells. The key stemness transcription factors were also consistently decreased by miR-203 and miR-200c (data not shown). Moreover, overexpression of miR-203 and miR-200c suppressed migration ability (Fig. 6c) and chemoresistance (Fig. 6d) in 16B/TNF cells. Collectively, these results indicate that restoration of miR-203 and miR-200c expression in 16B/TNF cells reverses the TNFα-induced CSC properties, suggesting that chronic TNFα exposure promotes cancer stemness of HOK-16B cells through downregulation of miR-203 and miR-200c.

Since activation of the Notch1 pathway is critical for the maintenance of CSCs,^42^ we explored whether the Notch1 pathway was activated in 16B/TNF cells by examining the expression of the activated form of Notch1 protein (NICD). The expression of NICD was significantly elevated in 16B/TNF compared to HOK-16B cells (lanes 1 and 2 in Fig. 6e). Furthermore, overexpression of miR-203 and miR-200c suppressed the expression of NICD in 16B/TNF cells (lanes 2–5 in Fig. 6e). To further confirm the activation of the Notch1 pathway, we examined the expression of Hes-1, a known NICD target gene (Fig. 6f). Hes-1 was also increased in 16B/TNF compared to HOK-16B and was suppressed by miR-203 and miR-200c in 16B/TNF cells. These findings indicate the activation of the Notch1 signaling pathway by chronic TNFα treatment in HPV-immortalized keratinocytes.

## Discussion

In this study, we propose that chronic inflammation promotes HPV-associated carcinogenesis by increasing cancer stemness. Chronic exposure of TNFα led to the acquisition of a malignant growth phenotype in HPV-immortalized keratinocytes, namely, (1) proliferation capacity at the physiological level of Ca^2+^, (2) anchorage-independent growth ability, (3) increased cell proliferation in vivo, and (4) self-renewal capacity. Moreover, TNFα increased the CSC population and properties in HPV-immortalized keratinocytes. Importantly, such TNFα effects were not observed in non-HPV-immortalized keratinocytes. Subsequently, our study revealed that HPV16 E6 played a key role in the TNFα-induced CSC phenotype via suppression of stemness-inhibiting miR-203 and miR-200c. Restoration of miR-200c and miR-203 reversed the TNFα-induced CSC properties in HPV-immortalized cells. Our findings provide evidence of the role of chronic inflammation in HPV-associated carcinogenesis and a mechanism through which chronic inflammation promotes HPV-associated carcinogenesis.

Previously, we reported successful immortalization of normal human oral keratinocytes by transfection with a high-risk HPV whole genome (i.e., HPV16 and HPV18), but this strategy failed to yield neoplastic conversion of normal cells.^8,9^ Our prior work suggests that HPV infection plays an important role in the early stage of oral carcinogenesis. However, the possible role of chronic inflammation in the malignant progression of HPV-immortalized cells, i.e., HOK-16B cells, has not been well documented.^43^ Many studies have reported the tumor-promoting effects of TNFα, a major mediator of inflammation. TNFα increased the malignant behavior of tumor cell lines.^44,45^ TNFα knock-out mice are resistant to chemically induced skin carcinogenesis.^46^ TNFα increased chromosomal instability by virtue of its ability to induce ROS.^47^ Similarly, our study clearly demonstrated that chronic exposure to TNFα induced a malignant growth phenotype in HPV-immortalized keratinocytes but not in non-HPV-immortalized keratinocytes, i.e., OKF6/tert cells. Moreover, chronic TNFα exposure induced greater chromosomal instability in HPV-immortalized keratinocytes than in non-HPV-immortalized keratinocytes (unpublished data). Previously, we reported that HPV-immortalized keratinocytes displayed impaired DNA repair activities.^48,49^ More specifically, the E6 and E7 oncoproteins of HPV16 interfered with the DNA repair process.^50,51^ Thus, we speculate that chronic TNFα may potentiate its mutagenic effect in HPV-infected cells with defective DNA repair capacity. Collectively, our data suggest that the chronic inflammatory microenvironment is an important additional factor for HPV-associated oral carcinogenesis.

Gaiotti et al.^24^ reported that TNFα increased the expression of HPV16 E6/E7 in HPV16-immortalized cervical keratinocytes. We demonstrated that acute and chronic TNFα exposure had no effect on viral gene expression in HPV16-immortalized oral keratinocytes, indicating different effects of TNFα on HPV expression in a different cellular context. Our study suggests that the acquired calcium resistance, malignant growth and stemness phenotype in 16B/TNF cells is independent of the overexpression of E6/E7 induced by TNFα in HPV16-immortalized oral keratinocytes.

Recent studies demonstrated that proinflammatory cytokines, including TNFα, increased the CSC population and properties in human cancer, suggesting a possible link between CSCs and inflammation.^32,34,52^ CSCs are considered the seed of cancer for their crucial roles in the malignant behavior of cancer cells, i.e., metastasis, drug resistance, and tumourigenicity. We demonstrated that chronic TNFα exposure endowed HPV-immortalized cells with self-renewal capacity, a key feature of CSCs, and concomitantly increased the expression of self-renewal transcription factors. TNFα also led to a robust increase in the CD44^high^/CD24^low^ CSC population. CD44^high^/CD24^low^ cells have been identified as CSCs in different types of cancer, including OSCC.^53^ CD44^high^/CD24^low^ cancer cells displayed higher self-renewal capacity and important CSC properties, such as migration, chemo-radioresistance, and tumourigenic potential compared to other subpopulations.^53^ TNFα also increases the migration ability and chemo-radioresistance of HPV-immortalized cells. Furthermore, we documented that chronic TNFα treatment results in activation of the Notch1 signaling pathway, a critical CSC maintenance pathway.^42^ Therefore, the increased CSC population and properties induced by TNFα in HPV-immortalized cells is also an important observation supporting the role of chronic inflammation in HPV-associated carcinogenesis and cancer progression. This observation suggests that chronic inflammation further promotes the malignant progression of HPV-immortalized oral keratinocytes by increasing cancer stemness. It is interesting to note that epithelial-mesenchymal transition (EMT) is considered the critical biological change for cytokine-induced CSCs.^32^ We observed increased EMT phenotypes, such as downregulation of genes involved in cell junctions (data not shown) and enhanced migration ability in TNFα-treated HPV-immortalized keratinocytes.

There is increasing evidence of the importance of miRNAs in the genesis and maintenance of CSCs.^38^ Many studies have identified various miRNAs affecting the CSC phenotype. For instance, miR-203 and the miR-200 family (miR-200a, 200b, 200c, 141, and 429) were downregulated in CSCs isolated from various cancer types, and their regulation could alter CSC markers and properties, indicating that they are CSC-inhibiting miRNAs.^39,40,54^ miR-203 suppresses CSCs by targeting Bmi-1.^39^ miR-200 members inhibit CSC self-renewal and properties by suppressing Notch signaling.^55^ Activation of Notch signaling has been implicated in CSCs in mammalian cancer.^42^ We found that miR-203 and miR-200 members were substantially decreased by chronic TNFα treatment in HPV-immortalized cells. Further analysis revealed that miR-203 and miR-200c were suppressed by TNFα in HPV16 E6-expressing but not in HPV16 E7-expressing OKF6/tert cells, indicating the requirement of E6 for TNFα-induced suppression. In our study, overexpression (restoration) of miR-203 and 200c resulted in suppression of self-renewal capacity, migration and chemoresistance in TNFα-treated HPV-immortalized cells, supporting their functional role in the TNFα-induced CSC phenotype. Furthermore, miR-203 and 200c inhibited Notch signaling in TNFα-treated HPV-immortalized cells. Therefore, we hypothesize that the exposure of HPV-infected cells to chronic inflammation generates a CSC population and allows CSCs to subvert immune-mediated elimination,^56^ thereby permitting long-term survival, which would promote the further oncogenic transformation of the cells.

It is interesting to note that E6 is known to inhibit the Notch pathway by downregulating Notch expression via inhibition of p53.^57^ Our study showed that chronic TNFα exposure activated the Notch pathway in HOK-16B cells. Together with the fact that E6 and E7 expression were not altered in 16B/TNF cells, our findings suggest that activation of the Notch pathway in 16B/TNF cells is independent of the E6/p53 axis. Moreover, we showed that ectopic expression of miR-203 and miR-200c suppressed the Notch pathway in 16B/TNF cells. Therefore, our findings indicate a novel activation mode of the Notch pathway *via* the TNFα/miR-203/miR-200c axis in HPV-immortalized oral keratinocytes.

Our findings also have strong implications for whether an HPV-infected keratinocyte promotes amplification of the HPV genome and propagation of the virus or blocks propagation of the virus, stabilizes the HPV genome and leads to cancer. This decision for the HPV-infected cell to yield virus or form cancer has not been adequately addressed or understood in the field. The relevant observations are the induced CSC properties and inability to differentiate when cells with the HPV genome are treated with TNFα. Keratinocytes must differentiate in order to amplify the HPV genome and ultimately yield viral particles. Therefore, it is possible that keratinocytes infected with HPV in the host will be shunted towards cancer formation instead of viral production if the cells persist in a strong inflammatory state.

In conclusion, this study provides novel information on the role of chronic inflammation in HPV-associated oral carcinogenesis. Our findings provide evidence for a novel synergism between chronic inflammation and HPV infection and a plausible mechanism underlying the cooperative effect of both critical cancer factors in carcinogenesis.

## Materials and methods

### Cell culture and chronic exposure of cells to TNFα

Two non-tumourigenic immortalized human oral keratinocyte cell lines (HOK-16B and OKF6/tert2) were cultured in Keratinocyte Growth Medium (KGM) (Lonza), as described previously.^8,58^ The HOK-16B cell line was established by transfecting normal human oral keratinocytes (NHOK) with the HPV-16 whole genome.^8^ The OKF6/tert cell line was established by transducing NHOK with hTERT.^58^ Both cell lines were treated with 5 ng·mL^−1^ TNFα (Sigma-Aldrich) for extended periods. SCC4, a human tongue squamous cell carcinoma cell line, was cultured in DMEM/F12 medium (Invitrogen), as described in our publication.^59^

### Anchorage-independent growth

To determine colony-forming efficiency in semi-solid medium, 1 × 10^4^ cells were plated in culture medium containing 0.3% agarose over a base layer of serum-free medium containing 0.5% agarose. The assay was performed as described previously.^59^

### In vivo tumourigenicity assay

Ten million cells were subcutaneously injected into the flanks of immunocompromised mice (strain *nu/nu*, Charles River Laboratories). The animal study was performed as described previously.^59^

### Tumor sphere formation assay

For tumor sphere formation, 4 000 cells were grown in 4 mL DMEM/F12 medium with 1:50 B27 (Invitrogen), 20 ng·mL^−1^ EGF, 10 μg·mL^−1^ insulin, penicillin, streptomycin, and amphotericin B in Ultra-Low Attachment 6-well Plates (Corning), as described in our previous study.^59^

### Quantitative real-time PCR (qPCR)

cDNA was synthesized by the SuperScript first-strand synthesis system (Invitrogen). The cDNA pool of miRNAs was synthesized by the QuantiMir cDNA Kit (System Biosciences) according to the manufacturer′s protocol. PCR amplification was performed with SYBR Green I Master Mix (Roche) on LightCycler 480 (Roche), as described previously.^59^ The primer sequences were obtained from the Universal Probe Library (Roche), and the sequences are available upon request.

### Flow cytometry

Cell surface expression of CD44 was determined by flow cytometry analysis on a FACScan (Becton Dickinson). Anti-CD44 FITC (BD Pharmingen) and secondary antibody FITC goat anti-rabbit IgG (Jackson ImmunoResearch Laboratories) were used.

### Migration assay

Cell migration was measured using transwell chambers with polycarbonate membranes (Corning) according to the method described in the manufacturer’s protocol and in our previous publication.^34^

### Chemo-radioresistance assays

Cisplatin and methotrexate were purchased from Sigma-Aldrich. Chemoresistance of cells was determined by measuring cell viability using the tetrazolium salt (MTT) cell proliferation assay kit (ATCC) as described elsewhere.^34^ For the radioresistance assay, the cells were exposed to ionizing radiation (IR) at varying doses using the Mark I-30 Cesium-137 irradiator (JL Shepherd & Assoc.,) with a delivery rate of 4.86 Gy ng·min^−1^ as described in our previous study.^34^

### Expression of HPV16 E6 and E7

Retroviral vectors capable of expressing HPV16 E6 or E7 were constructed from pLXSN plasmids as described in our previous publication.^51^ Retroviruses expressing the empty vector or a viral gene were harvested from the transfected PA317 cells. Forty to 60 percent confluent OKF6/tert cells were infected with the retroviruses. After the infection, the cells were cultured in medium containing 100 μg·mL^−1^ G418 (Invitrogen). Then, G418-resistant clones were collected and used for the experiments.

### Western blotting

Western blotting was performed as described previously.^59^ We used the following primary antibodies for this study: anti-p53 (Oncogene Science), anti-NICD (Cell Signaling Technology), anti-actin (Santa Cruz Biotech) and anti-GAPDH (Santa Cruz Biotech).

### Overexpression of microRNA

Cells were transfected with pre-miR negative control (#AM17110; Ambion), pre-miR-200c (Ambion), or pre-miR-203 (Ambion) at a final concentration of 50 pmol using Lipofectamine 2000 (Invitrogen) according to the manufacturer's protocol.
